# Influence of biochar on the removal of Microcystin-LR and Saxitoxin from aqueous solutions

**DOI:** 10.1038/s41598-024-61802-z

**Published:** 2024-05-14

**Authors:** Cadianne Chambers, Savannah Grimes, Spencer Fire, M. Toufiq Reza

**Affiliations:** 1 Department of Chemistry and Chemical Engineering, Florida Institute of Technology, Melbourne, FL 32901 USA; 2 Department of Ocean Engineering and Marine Sciences, Florida Institute of Technology, Melbourne, FL 32901 USA

**Keywords:** Harmful algal blooms, Microcystin-LR, Saxitoxin, Biochar, Adsorption equilibrium, Adsorption kinetics, Chemical engineering, Pollution remediation

## Abstract

The present study assessed the effective use of biochar for the adsorption of two potent HAB toxins namely, Microcystin-LR (MCLR) and Saxitoxin (STX) through a combination of dosage, kinetic, equilibrium, initial pH, and competitive adsorption experiments. The adsorption results suggest that biochar has excellent capabilities for removing MCLR and STX, with STX reporting higher adsorption capacities (622.53–3507.46 µg/g). STX removal required a minimal dosage of 0.02 g/L, while MCLR removal needed 0.4 g/L for > 90%. Similarly, a shorter contact time was required for STX removal compared to MCLR for > 90% of toxin removed from water. Initial pH study revealed that for MCLR acidic conditions favored higher uptake while STX favored basic conditions. Kinetic studies revealed that the Elovich model to be most suitable for both toxins, while STX also showed suitable fittings for Pseudo-First Order and Pseudo-Second Order in individual toxin systems. Similarly, for the Elovich model the most suited kinetic model for both toxins in presence of each other. Isotherm studies confirmed the Langmuir–Freundlich model as the best fit for both toxins. These results suggest adsorption mechanisms including pore filling, hydrogen bonding, π–π interactions, hydrophobic interactions, electrostatic attraction, and dispersive interactions.

## Introduction

For centuries, harmful algal blooms (HABs) have been polluting water bodies and have frequently caused harm to both human and aquatic life in U.S. coastal waters^1^. These blooms, (and associated release of natural toxins) are increasingly occurring due to excessive nutrient loading from anthropogenic activities and climate change^2^. As a result, HABs cause significant negative environmental impacts, resulting in economic losses for aquaculture, fisheries, tourism, and human healthcare^3^. HAB toxins can also cause serious ailments in humans, sometimes resulting in fatalities^4,5^. Given the severe consequences of exposure to such toxins, the treatment of these affected waters is of paramount importance.

A wide variety of HAB toxins have been reported in fresh and brackish waters. Cyanobacteria blooms (“blue-green algae”), in freshwater systems have been studied extensively due to the wide catalog of toxins they produce^6^. The cyanotoxins produced during these blooms have been documented to cause neurological, gastrological, dermatological, and hepatic symptoms in humans^7^. Microcystins (MC), a well-studied class of cyanotoxins, are natural hepatotoxins released during cyanobacteria blooms that frequently impact aquatic ecosystems^8^. This raises concern as the reoccurring nature of these blooms might lead to prolonged exposure for affected individuals. For example, in response to high levels of rainfall, large quantities of water are commonly discharged from Lake Okeechobee to the St. Lucie Estuary in Florida, resulting in intense annual summer blooms of *Microcystis aeruginosa*^9^. These events halt operations in these areas, prompting organizations like the U.S. Environmental Protection Agency (EPA) to issue health advisories recommending against consumption and swimming in contaminated waters^10^.

Another concerning algal toxin produced by cyanobacteria is Saxitoxin (STX), belonging to a class of potent neurotoxins that causes Paralytic Shellfish Poisoning (PSP) in humans^11^. STX is produced not solely by freshwater cyanobacteria but also from marine dinoflagellates species like *Alexandrium spp.*, *Gymnodium catenatum*, and *Pyrodinium bahamense var compressum*^9,12^. Thus, the risk of STX exposure exist in both fresh and brackish water habitats^13^. Elevated concentrations of STX (3–8 µg/L) have been reported in Florida’s Indian River Lagoon (IRL), above the EPA regulatory limit of 0.2 µg/L^14^. This elevation could be made possibly due to warmer waters and/or increased nutrient loading^15^. Since various phytoplankton species are known producers of STX, the occurrence of both STX and MC being reported frequently in the same body of water poses significant threats to the environment. For instance, high levels of MC and STX have been recorded across the IRL, ranging between 0.01 and 85.70 μg/L and 0.01 and 2.43 μg/L, respectively^9^. Therefore, there is an urgent need to explore mitigation practices suitable for addressing HABs that produce these toxins in this region.

In recent review studies, some current techniques used to reduce these cyanotoxins have been reported in recent publications. These techniques involve the use of membranes (including nano- and ultrafiltration), potassium permanganate, ozonation, UV radiation, free chlorine, and physical adsorption utilizing carbon-based adsorbents^16^. However, there have been shortcomings with the use of some of these techniques, as certain variables present negative factors for the removal process. For example, chemical processes are only recommended as a last resort option as the negative aspects outweigh the positives, due to (1) proliferation of lysing algal cells, (2) potential harm to the aquatic ecosystem, and (3) the potential of introduction of novel toxins effects to aquatic organisms^16,17^. Additionally, chemical process such as advanced oxidation has proven to be an excellent upcoming mitigation technique for the degradation of Microcystin-LR through the use of novel photocatalysts^18,19^. While superior degradation efficiency has been reported for larger hydrophobic molecules like Microcystin-LR similar performance is not shown for smaller hydrophilic molecules like Anatoxin-a and Saxitoxin^20,21^. This indicates that certain techniques are effective in treating specific cyanotoxins but may not be universally effective across all types. Adsorption process have been the stand-alone solution, as high removal efficiency, affordability, and design simplicity for the removal of cyanotoxins have proven to be the most advantageous compared to other treatment alternatives^22–24^. This process would be applied in field as a supplementary treatment to the dissolved extracellular toxins as envisioned in full/pilot scale studies as supported by literature^25–27^. This is due to the fact that while mitigation strategies such as coagulation/flocculation, sedimentation, dual media filtration, chlorination and ozonation are efficient against the intracellular cells, for drinking water treatment plants (DWTPs) additional treatment is required^27^. Interestingly, adsorption methods have been instrumental in the control strategies regarding HAB via Solid Phase Adsorption Toxin Tracking (SPATT) bags which use porous synthetic resins capable of adsorbing toxins directly from the water column^28^. This practical use has been utilized in field reporting sensitive detections of domoic acid, saxitoxins, anatoxins and microcystins^28,29^. Thus, a viable adsorbent material prioritizing the effective selective adsorption of cyanotoxins presents as a valuable course of action in the mitigation of these blooms.

Biochar, a pyrogenous material derived from plant or animal feedstock under an oxygen-limiting environment, has shown excellent potential as a cost-effective adsorbent technology^30^. Presenting as a carbon-dense, highly porous, and functional material^30^. These physicochemical properties vary depending on the feedstock and can be employed for various applications, including soil remediation and amelioration, carbon sequestration, wastewater decontamination, electrode materials, catalysts and more^31,32^. Focusing on water purification, biochar has found wide usage in the adsorption of contaminants like heavy metals, microplastics, and nutrients,^33,34^. Consequently, this application has extended to the exploration of using physical adsorbents like biochar for mitigating HAB toxins^35–40^. For instance, Wei et al. explored rice straw derived biochars synthesized at different pyrolysis temperature for the adsorption of MCLR reporting maximum adsorption capacity of 10.96 µg/g^35^. Zeng et al. studied the utilization of novel iron activated biochars derived from pyrolysis and chemical activation of bermudagrass removing MCLR in aqueous solution, reporting adsorption capacity ranging between 760 and 9000 µg/g^36^. Song et al. presented Kentucky bluegrass-derived biochar effectively adsorbed Microcystin-LR (MCLR) with maximum adsorption capacity of 2,769 µg/g, aided by a complex of various adsorption mechanisms^37^. Hydrophobic contacts, π–π interactions, electrostatic attraction, ion exchange, and surface complexation of biochar are thought to play dominant roles for the uptake of MCLR^37^. Again, these findings support the cost efficiency of biochar as a low-cost advanced treatment as the selling price of biochar ranges from 0.35 to 1.2 US$/Kg an approximation of 2.9–10 g of toxin being adsorbed per 1 US$ based on the high adsorption capacity^22^.

In the interest of using similar adsorbents, Melegari et al. evaluated the use of natural adsorbents, such as chitin and oyster powder, for the adsorption of Saxitoxin in water^38^. The study indicated that adsorption was favorable, ranging between 0.0446 and 0.06424 µg/g for chitin and 0.04665 and 0.05470 µg/g for oyster powder^38^. Buarque et al. reported high removal efficiency with the using coconut derived activated carbon, with adsorption capacities ranging between 252 and 3034 µg/g^39^. The pH impact on STX adsorption with activated carbon was studied by Shi et al., which identified the maximum adsorption ranging between 270 and 12,980 µg/g^40^. Evidently, at higher pH, the electrostatic attraction showed a dominant influence on STX adsorption because of the attraction between the positively charged toxin and the negatively charged adsorbent surface^40^. However, there still appears to be limited research utilizing biochar for STX removal in aqueous solutions.

While considerable amount of literature has proven carbon-based adsorbents capability in adsorbing MCLR and STX independently, it is imperative to shed light on how varied adsorption conditions influences uptake of these toxins. This requires investigating conditions such as adsorbent dosage, contact time, initial concentration of adsorbate, and initial pH^41,42^. These findings have the potential to enhance adsorption efficiency while elucidating the underlying adsorption mechanisms involved as contact time results provide insight on the adsorption kinetics^43^ and initial concentration reveals the adsorption isotherms involved^44^. For instance, for biotoxin studies, contact time remains an important adsorption parameter as over extended contact time (> seven days) can result to toxin loss due to desorption for hydrophilic toxins in water column studies^28^. Although these parameters have been studied for both MCLR and STX, there remains a limited understanding of how these conditions respond differently to the dual use of biochar. For biochar to remain a state-of-the-art technology, high selectivity is important as these toxins can coexist with other contaminants including nutrients (anions/cations), dissolved organic matter (humic/fulvic acid), and additional cyanotoxins in varying waterbodies^45–51^. For this study, the influence of the combined presence of differing cyanotoxins such as MCLR and STX would provide insightful findings on the competitive adsorption with biochar as both can exist together naturally in fresh waterbodies. Compared to the other contaminants, there is still little information regarding the temporal competition among these toxins. Thus, addressing these knowledge gaps will prove beneficial to rehabilitation of HAB waters.

**Figure 1:**
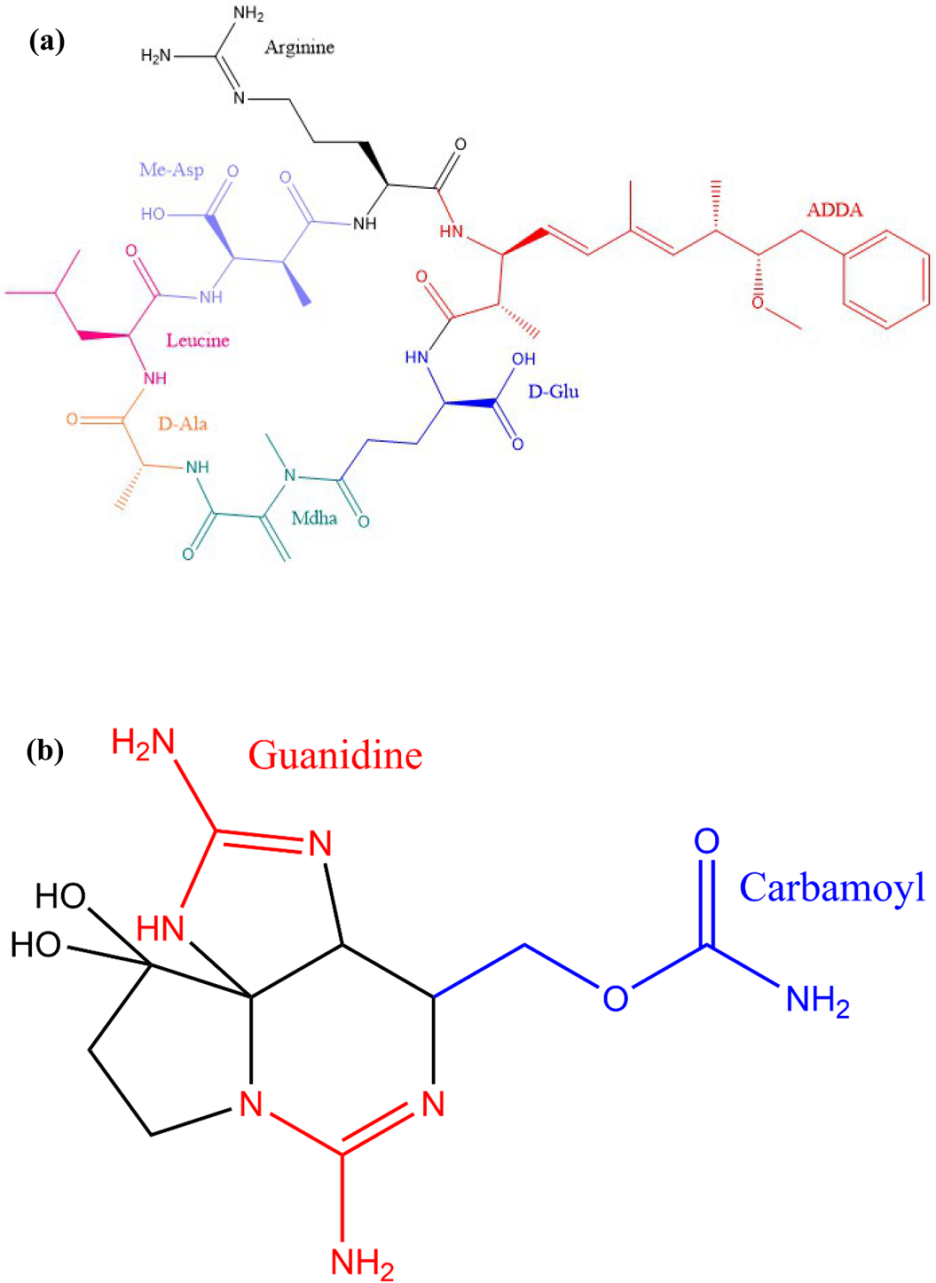
Chemical structure of (**a**) Microcystin-LR (MCLR) and (**b**) Saxitoxin (STX).

This work evaluated the adsorptive capabilities of biochar in order to remove MCLR and STX from experimental solutions. MCLR and STX were chosen as congeners of cyanobacteria for this study. MCLR is one of the most significant algae toxins in the United States due to its potent toxicity^52,53^, while STX is the most common and toxic congener of Paralytic Shellfish toxins (PSTs)^54^. It is important to highlight the difference in the physicochemical characteristics of MCLR and STX, reported in Table 1 and Fig. 1, based on literature^55–58^. From observations, MCLR is a larger molecule with higher hydrophobicity and lesser toxicity compared to STX. This poses a serious threat, considering STX is highly toxic, requiring only 300 μg to fatally poison humans^59^. This threat enforces the imperative search in mitigating these toxins, which occur together at threatening levels. In this study, adsorption parameters including dosage rate, contact time, initial concentration, and initial pH were investigated to gauge the biochar efficacy as a suitable adsorbent for both toxins. In addition, biochar capability of simultaneous adsorption of both toxins was assessed under optimal adsorption parameters. In short, batch adsorption testing was implemented to assess biochar efficiency in adsorbing MCLR and STX using the previously mentioned adsorption parameters. These results yielded exceptional results regarding adsorption capacity, modelling behavior (isotherm and kinetic models), which were comparable to literature. In addition, these investigations of biochar showed excellent removal efficiency towards both toxins, highlighting various adsorption mechanisms possible for the removal. In evaluating the adsorption affinity for these target HAB toxins, the data obtained proved to be critical to the long-term goal of establishing effective mitigation strategies to the environmental threats posed by HABs.

**Table 1:** Selected physicochemical properties of Microcystin-LR (MCLR) and Saxitoxin (STX)^55–58^.

HAB Toxin	Microcystin-LR (MCLR)	Saxitoxin (STX)
Molecular formula	C_49_H_74_N_10_O_12_	C_10_H_17_N_7_O_4_
Molecular weight	995.2 g/mol	299.29 g/mol
Density	1.29 g/cm^3^	1.369 g/cm^3^
Solubility in water	7.02 × 10^–6^ g/g	0.01 g/g
LC_50_^a^	18 mg/m^3^	0.3 mg/m^3^

^a^Toxicity tested against a mouse via inhalation.

## Materials and methods

### Materials and chemicals

Pine-derived biochar was procured from Green Carbon Solutions, (FL, USA). Biochar underwent an overnight drying process at 105 °C to remove any remaining moisture. After which, the biochar was pulverized using a mortar and pestle to a fine powder (500 µm). The resultant biochar was gathered and kept in vials. The following items were purchased from Fisher Scientific for material characterization, 0.01 N sodium hydroxide (NaOH), 0.01 N hydrochloric acid (HCl), and potassium nitrate (KNO_3_) (99%. For adsorption experiments, methanol (99%) was purchased from Fisher Scientific, while both MCLR (95% purity) and STX (95% purity) standards were procured from Gold Standard Diagnostics (Davis, California). Biochar characterization methods and results are presented in supplementary information. For toxin quantification, Enzyme-Linked Immunosorbent Assay (ELISA) kits were also acquired from Gold Standard Diagnostics (Davis, California).

### Adsorption methodology

#### Adsorption experiments

Batch adsorption studies were carried out to investigate how biochar interacted with MCLR and STX solutions. Various adsorption parameters were systematically examined to assess the impact on adsorption efficiency. These include dosage amount, contact time, initial concentration, and initial pH of the toxin. The dosage analysis study, biochar was subjected to varying dosage rates (0–1 g/L) and agitated with 10 µg/L of MCLR and STX solutions for 24 h. The contact time study involved varying the agitation time (0.5–48 h) of biochar with 50 µg/L MCLR and STX solutions at optimal dosage rate. The initial concentration study focused on different concentrations (10–100 μg/L) of MCLR and STX solutions, agitated with biochar at the optimal dosage rate and contact time. For the initial pH study, pH range of 2–10 with an initial concentration of 50 µg/L of MCLR and STX were utilized*.* This variation was investigated with biochar at the optimal dosage rate and contact time. In the competitive adsorption study, conducted as a contact time study, a binary-component system containing an equal concentration of 50 µg/L of MCLR and STX. All adsorption experiments were duplicated to assess reproducibility. To minimize vial headspace, 11.5 mL of toxin solution was added to 12 mL amber glass vials, preventing loss from volatilization. In parallel, blank runs excluding biochar, were performed to each study to evaluate toxin loss due to sorption onto the vials. All experiments were conducted at ambient temperature in the absence of light to prevent toxin degradation through photolysis. Once adsorption is complete, the remaining toxin is filtered through 0.22 µm Nylon syringe-driven filters, and subsequent analysis was carried out to detect the final concentration.

#### Adsorption analysis

MCLR and STX concentrations were measured using ELISA kits due to it high selectivity and accuracy for detection^60^. The observed range of detection for both MCLR and STX were 0.15–5 and 0.02–0.4 µg/L respectively, as such, if necessary, dilutions were performed for detection. The MCLR and STX percent removed and adsorption capacity, q_e_ (µg/g) of the biochar were evaluated using Eqs. (1) and (2) respectively.

1 $$ {\text{Percent Removed}} = \frac{{{\text{C}}_{0} - {\text{C}}_{{\text{e}}} }}{{{\text{C}}_{0} }} \times 100\% $$

2 $$ {\text{q}}_{{\text{e}}} = \frac{{({\text{C}}_{0} - {\text{C}}_{{\text{e}}} ) \times {\text{V}}}}{{\text{m}}} $$

where the initial and final MCLR and STX concentrations (µg/L) are denoted by C_0_ and C_e_, respectively, solution’s volume is V (L), and the mass of the biochar is m (g).

#### Adsorption models

Using non-linear regression analysis, the isotherm models applied were Langmuir Model, Freundlich Model, and Langmuir–Freundlich Model (Eqs. 3, 4, and 5), respectively. Non-linear regression was performed using OriginPro 2022 Version 9.90 (https://www.originlab.com/2022), which provided all models converging at the 0.05 level of significance.

3 $$ {\text{q}}_{{\text{e}}} { = } \frac{{{\text{q}}_{{{\text{mL}}}} \cdot {\text{K}}_{{\text{L}}} \cdot {\text{C}}_{{\text{e}}} }}{{{1 + } {\text{K}}_{{\text{L}}} \cdot {\text{C}}_{{\text{e}}} }} $$

4 $$ {\text{q}}_{{\text{e}}} = {\text{K}}_{{\text{F}}} \cdot {\text{C}}_{{\text{e}}}^{{1/{\text{n}}_{{\text{f}}} }} $$

5 $$ {\text{q}}_{{\text{e}}} { = }\frac{{{\text{q}}_{{{\text{mLF}}}} {(} {\text{K}}_{{{\text{LF}}}} \cdot {\text{C}}_{{\text{e}}} {)}^{{\upbeta }} }}{{{1 + } {(} {\text{K}}_{{{\text{LF}}}} \cdot {\text{C}}_{{\text{e}}} {)}^{{\upbeta }} }} $$

For Langmuir, the maximum adsorption capacity is q_mL_ (µg/g), the equilibrium constant, K_L_ is related to free energy (L/µg); for Freundlich, equilibrium constant, K_F_ is related to the relative adsorption capacity (L/mg) and n_f_ is the intensity of the adsorption ; finally for Langmuir–Freundlich, q_mLF_ is the maximum adsorption capacity (µg/g), K_LF_ is the equilibrium constant for a heterogenous solid, and β is the heterogeneity parameter (0–1)^61^.

Equations 6, 7 and 8 illustrated the kinetic models applied to the adsorption data; Pseudo-First Order, Pseudo-Second Order and Elovich Model using non-linear regression respectively.

6 $$ {\text{q}}_{{\text{t}}} { = } {\text{q}}_{{\text{e}}} \left( {{1} - {\text{e}}^{{ - {\text{k}}_{{1}} {\text{t}}}} } \right) $$

7 $$ {\text{q}}_{{\text{t}}} { = }\frac{{{\text{q}}_{{\text{e}}}^{{2}} \cdot {\text{k}}_{{2}} \cdot {\text{t}}}}{{{\text{1 + q}}_{{\text{e}}} \cdot {\text{k}}_{{2}} \cdot {\text{t}}}} $$

8 $$ {\text{q}}_{{\text{t}}} { = } \frac{{1}}{{\text{b}}}{\text{ln}}\left( {\text{1 + abt}} \right) $$

where, q_t_ is the adsorption capacity at time t (µg/L), t is time, k_1_ is the pseudo-first order rate constant (min^−1^), k_2_ is the pseudo-second order rate constant (g·μg^−1^ min^−1^), a is the initial adsorption rate constant of the Elovich model (μg·g^−1^ min^−1^) and b is the desorption rate constant of the Elovich model (g µg^−1^)^62^. The determination coefficient (R^2^) and the residual mean square error (RMSE) were used to which assess the adsorption models suited the data best.

## Results and discussion

### Effect of biochar dosage on adsorption of MCLR and STX

**Figure 2:**
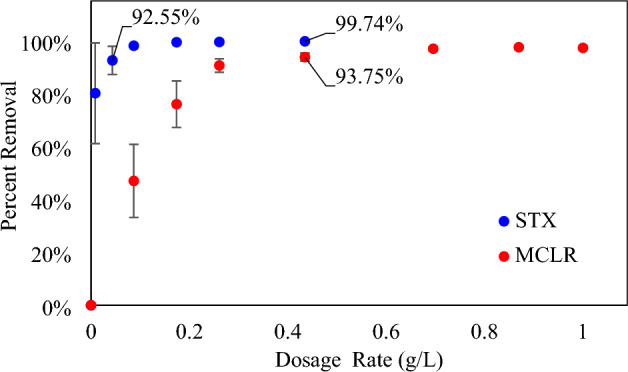
Dosage Study results for biochar with Microcystin-LR (MCLR) and Saxitoxin (STX).

From the dosage study, biochar independently in contact with both MCLR and STX exhibited high percent removal with incremental increase in dosage amount up to constancy after a certain amount (Fig. 2). This phenomenon is explained by the fact that the surface area and number of adsorbent active site increase with increase in adsorbent supplied^63,64^. For MCLR, a minimal dosage rate of 0.4 g/L of biochar is required for > 90% toxin removal, while STX, requires only 0.04 g/L. It is noteworthy that dosage rates exceeding 0.4 g/L rendered the final concentration of STX below the limit of detection (0.01 μg/L). Thus, at smaller doses of biochar, high percentage removal is attainable for STX in comparison to MCLR. This behavior can be attributed to STX being a smaller molecule (241–491 Daltons^65^) than MCLR (800–1100 Daltons^65^), which can facilitate more adsorption mechanisms. Regardless, the dosage rate of biochar at 0.4 g/L showed excellent removal efficiency for both MCLR and STX*,* which was maintained for contact time study.

### Effect of contact time on MCLR and STX adsorption on biochar

**Figure 3:**
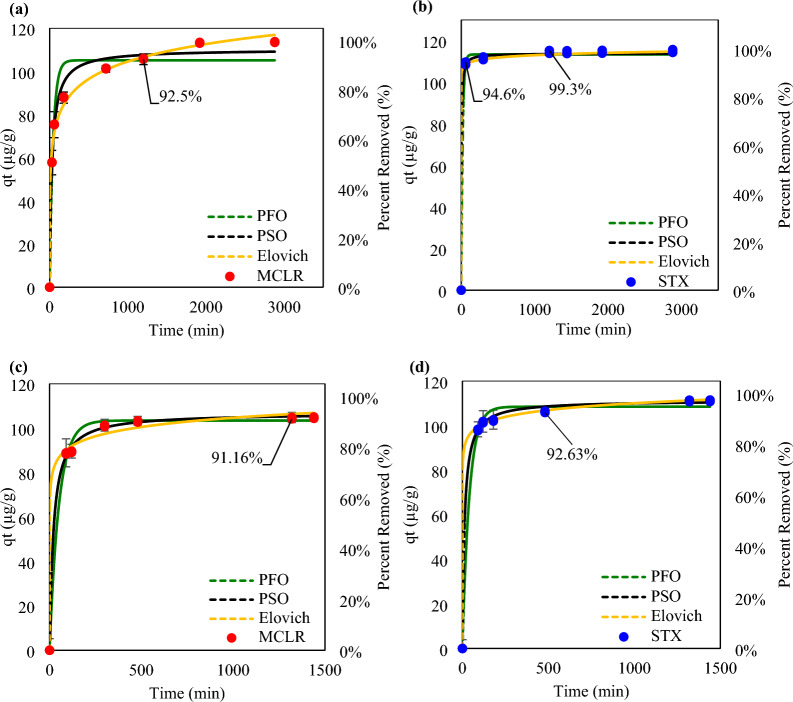
Kinetic Study results for biochar in a single-component system (**a**) Microcystin-LR (MCLR), (**b**) Saxitoxin (STX) and binary-component system of (**c**) Microcystin-LR (MCLR) and (**d**) Saxitoxin (STX).

For the contact time study, increasing the contact time between the biochar and both MCLR and STX independently led to a higher percentage of toxin removal (Fig. 3a–b). At which equilibrium was met at the 20th h for MCLR and at the 5th h for STX. Thus, contact time of 20 h or more provides sufficient time for both toxins to attain favorable adsorption efficiency with biochar. The increase in uptake with the increase in time is typical as the toxins are allowed more time to adhere to the biochar as interactions. To attain > 90 of toxin removal it can be observed that MCLR requires longer contact time of 20 h compared to STX which only requires 1 h. This suggests that STX participates in a rapid adsorption process with biochar while MCLR partakes in a slower process to attain high removal efficiency. This initial rapid adsorption is explained by Pavagadhi et al.^47^, where easy diffusion through pores is engaged by the available chemical active sites, increasing the concentration gradient between the toxin in solution and toxin in the biochar. Strong attraction forced between the toxin ant biochar propels rapid diffusion through the intraparticle matrix and bringing to a quick equilibrium^47^. Again it is important to highlight that STX is a smaller molecule than MCLR, therefore it is capable of performing easy pore diffusion while MCLR may face limitations on diffusion depending on the orientation of molecule^66^. Slower adsorption processes are usually explained by the chemical reactions involving the adsorbent and adsorbate (chemisorption)^67^. Thus, STX rapid adsorption corresponds more to a physical adsorption while MCLR slow adsorption corresponds to a chemical adsorption. When toxins are in the presence of each other (Fig. 3c–d), a longer contact time is required to attain > 90% removal as MCLR needed 22 h and STX requires a 8 h. This delay in removal efficiency can be due to hinderances in the adsorption mechanisms due to dual presence of each toxin. Nevertheless, the trend remains the same as MCLR took a longer time than STX in achieving high percent removal.

### Effect of initial concentrations of MCLR and STX on their adsorption on biochar

**Figure 4:**
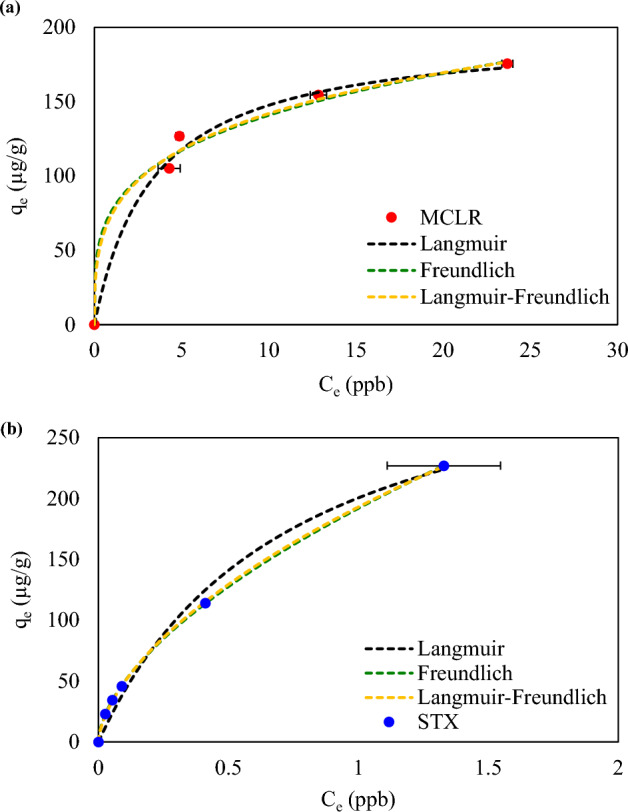
Equilibrium Study results for biochar with (**a**) Microcystin-LR (MCLR) and (**b**) Saxitoxin (STX).

For the initial concentration study, using optimized dosage rate and contact time parameters high adsorption capacities were observed for the HAB toxins (Fig. 4). When the initial concentration increased, the adsorption capacities of MCLR and STX both increased. This behavior is initiated as the increase in initial concentration which increases the adsorption capacity by overriding the mass transfer resistance of the molecules from the aqueous phase to the solid phase^35,68^. In comparing each toxins adsorption capacities with biochar, MCLR showed up to 176.35 µg/g while STX displayed higher adsorption capacity up to 226.94 µg/g. This higher performance in STX uptake could be based on the advantage STX has as revealed by both the dosage and contact time studies. As revealed, the applied dosage amount of 0.4 g/L would be exceeding advantageous for higher uptake as more than required amount is accessible allowing higher removal efficiency than MCLR. The contact study also revealed, STX depicting both physical and chemical adsorption, which may also aid in the out performance of MCLR adsorption. Nevertheless, high uptake is observed for both MCLR and STX in contact with biochar, validating the effective usage of the adsorbent as component in HAB mitigation strategies.

### Effect of initial pH on MCLR and STX adsorption on biochar

**Figure 5:**
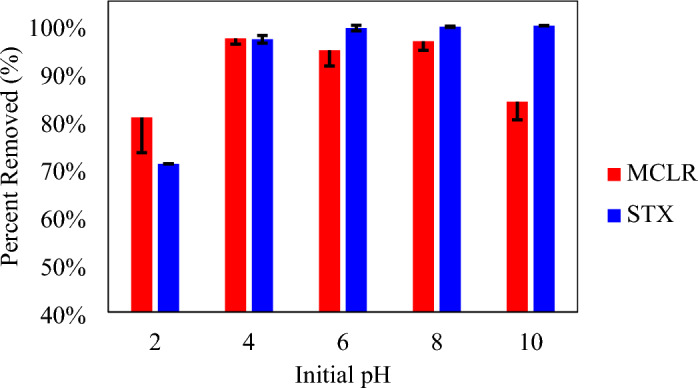
Initial pH Study results for biochar with Microcystin-LR (MCLR) and Saxitoxin (STX).

For the initial pH study, optimized dosage rate and contact time were used in the variations of initial pH of 2–10 supplied efficient toxin removal (Fig. 5). Increases in pH caused percent removed for MCLR to rise then decline, whereas increases in pH caused percent removed for STX to rise. For > 90% toxin removal, an initial pH of 4–8 was required with pH 4 exhibiting the highest percentage removed (97.92 ± 0.80%) for MCLR. On the other hand, an initial pH 4–10 was required, with pH 10 reporting the highest percentage removed (99.85 ± 0.03%) for STX. From the characterization results, the investigated biochar possesses a positive surface charge under neutral to acidic conditions (pH_PZC_ = 8.33). This consequently facilitates charge attraction or repulsion between the toxin and adsorbent. The MCLR molecule contains carboxylic acid groups and amino acid groups, imparting a negative charge at pH values 3 and 12^69^. This explains the higher removal efficiency at lower pH, as electrostatic attraction aids adsorption, while at higher pH, repulsion ensues between the toxins and biochar, limiting uptake. This behavior aligns with existing literature, owing to electrostatic attraction between the microcystin and the adsorbent^46,70–72^. In addition, reports have expressed the behavior of microcystin to cluster together and decrease in size under acidic conditions, allowing the toxin to be adsorbed onto the adsorbent more effectively^71,73^. The STX contains several amine groups which potentially protonate depending on the pH^40^. Shi et al. provided a detailed analysis on the differing charge of STX at varying pH, stating that at pH < 9, it possess a positive charge, at pH between 9–12, it holds a neutral charge, and at pH > 12 it claims a negative charge^40^. This rationalizes the trend of increasing removal efficiency with increasing pH, as electrostatic repulsion is lessened up to pH 10, where repulsion disappears. This negates the electrostatic attraction mechanism aiding in adsorption. However, these results are similar to literature which observe high adsorption between pH 9 and 12 due to non-electrostatic interactions^40^.

### Competitive adsorption of MCLR and STX adsorption on biochar

For the competitive adsorption study, binary-component system using [MCLR]_0_ = [STX]_0_ = 50 μg/L was tested against optimized dosage rate and initial pH of 5. There was only a recognized decrease in adsorption capacity at equilibrium of 7.70% for MCLR and 2.53% for STX. This indicates while there may be hinderances in the removal of each toxin, the biochar still showed promising percent removal in the removal of both toxins in the presence of each other. From literature, competitive adsorption takes place when the adsorption of one of the adsorbates is greatly affected due to the presence of the other adsorbate^74^. Thus, the competition between MCLR and STX proved to be slight as the adsorption capacity was not greatly reduced. It has been accounted that competitive adsorption can be led by adsorbates of similar molecular sizes or adsorbates sourcing similar active adsorption sites^75^. Since MCLR and STX are magnitudes different in molecular size, this could account for the low competition in adsorption when in the presence of each other. In addition, as detailed earlier MCLR and STX molecules may target different active sites due to the difference in surface charge and pore structure. Hence, this could also justify the low adsorption competition. This is comparable to another study by Rorar et al. that demonstrated the removal of Saxitoxin and Anatoxin-a using powdered activated carbon (PAC) in both the presence of absence of Microcystin-LR and/or Cyanobacterial cells^49^. It was determined that the presence of Microcystin-LR enhanced the removal of STX*,* while simultaneously removing both toxins at investigated initial concentrations of 1.6 μg/L and 20 μg/L at varied pH^49^. As for the competitive adsorption of STX and MCLR cells, the removal efficiency was slightly affected since at pH levels 6, 7 and 9 the values were 45%, 46%, and 65% for MCLR and 47%, 51% and 47% for STX.

### Adsorption isotherm modeling of MCLR and STX adsorption on biochar

The findings of the applied isotherm models, shown in Table 2, indicated that the Langmuir and Freundlich model are appropriate isotherms for both MCLR and STX (R^2^ > 0.99, low RMSE). This implies that both monolayer and multilayer behaviors between the toxin and the biochar might be taking place during the adsorption process. Thus, the Langmuir–Freundlich model was explored which well supports both adsorption behaviors with R^2^ > 0.99 and low RMSE. The Langmuir–Freundlich model indicates the heterogenous nature of the surface which supports the Freundlich model at low concentrations while the Langmuir model at high concentration^76^. The Langmuir–Freundlich model estimated the maximum adsorption capacity of MCLR and STX to be 622.23 and 3507.46 µg/g, respectively. Higher adsorption capacity is observed for STX with biochar which may be due to the variation of both physical and chemical adsorption mechanisms responsible for uptake.

**Table 2:** Adsorption Isotherm models fit with batch adsorption of biochar with Microcystin-LR (MCLR) and Saxitoxin (STX).

Adsorption isotherm models	Microcystin-LR (MCLR)	Saxitoxin (STX)
Langmuir model		
q_mL_ (μg/g)	210.23	376.84
K_L_ (µg/L)	0.22	1.14
R^2^	1.00	1.00
RMSE	6.31	0.004
Freundlich model		
K_F_ (L/µg)	78.23	192.02
n_F_	3.88	1.70
R^2^	1.00	1.00
RMSE	3.27	9.12 × 10^–6^
Langmuir Freundlich model		
q_mLF_ (μg/g)	622.53	3507.46
K_LF_	0.0028	0.0097
β	0.34	0.61
R^2^	1.00	1.00
RMSE	1.60	4.07 × 10^–6^

### Adsorption kinetics modeling of MCLR and STX adsorption on biochar

For the MCLR and STX contact time study with biochar, kinetic models including Pseudo-First order (PFO), Pseudo-Second order (PSO), and the Elovich model were used as shown in Table 3. These models elucidate the possible physisorption and chemisorption mechanisms occurring between the biochar and the toxins. The Pseudo-First order model, which proposes physisorption (diffusion) controls the rate of adsorption, is applicable for the first phase of the adsorption process, while Pseudo-Second order, is applicable to the entire adsorption process, which suggests chemisorption (chemical reaction) drives the adsorption rate^77,78^. Similarly, the Elovich model proposes mechanisms involving chemical interactions within the system which consisting of heterogenous adsorbing surfaces^79^.

**Table 3:** Kinetic models fit for the adsorption of biochar in single and binary-component system of *Microcystin-LR (MCLR)* and *Saxitoxin (STX)*.

Kinetic Models	Microcystin-LR (MCLR)		Saxitoxin (STX)	
	Single Component	Binary Component	Single Component	Binary Component
Pseudo-first order model				
q_e_ (μg/g)	105.16	103.46	113.39	108.48
k_1_ (min^−1^)	0.02	0.02	0.05	0.02
R^2^	0.96	1.00	1.00	0.99
RMSE	72.15	6.71	1.62	9.18
Pseudo-second order model				
q_e_ (μg/g)	110.28	106.90	113.87	111.33
k_2_ (g ug^−1^ min^−1^)	3.09 × 10^–4^	4.74 × 10^–4^	2.70 × 10^–3^	7.29 × 10^–4^
R^2^	0.99	1.00	1.00	1.00
RMSE	19.81	1.62	0.80	1.99
Elovich model				
a (μg g^−1^ min^−1^)	89.86	2.18 × 10^5^	2.65 × 10^27^	3.06 × 10^8^
b (g/µg)	0.09	0.17	0.62	0.23
R^2^	0.99	0.99	1.00	1.00
RMSE	12.16	7.48	0.12	0.55

For the contact study performed with biochar independently with MCLR and STX, the Elovich model was the best fit as shown in Table 3 with R^2^ > 0.99 and low RMSE for both toxins. However, it is important to note that STX also showed good, modeled relationships for the Pseudo-First and Pseudo-Second Order models (R^2^ > 0.99 and low RSME), thus indicating these adsorption behaviors. These results coincide with earlier discussion, as rapid adsorption was observed for the uptake of STX which can be linked to physically driven adsorption. In addition, a slower adsorption process was reported for MCLR which has been concurrent to a chemically driven adsorption process. Therefore, the Pseudo-First order is faster than Pseudo-Second order and Elovich model. Existing literature have concurred these adsorptions kinetics models of MCLR and STX on biochar^36,37,39,80^.

For the contact study performed using the binary system of MCLR and STX, the Pseudo-Second order model best fit the MCLR results, while Elovich model best fit the *STX* results (R^2^ > 0.99 and low RMSE). Both models show strong influence of chemisorption interactions between the toxin and biochar which enable removal. This further concludes initial discussion, as the uptake of both toxins in the presence of each other occurs slowly, thus indicating chemical driven adsorption response.

### Proposed adsorption mechanisms of MCLR and STX adsorption on biochar

**Figure 6:**
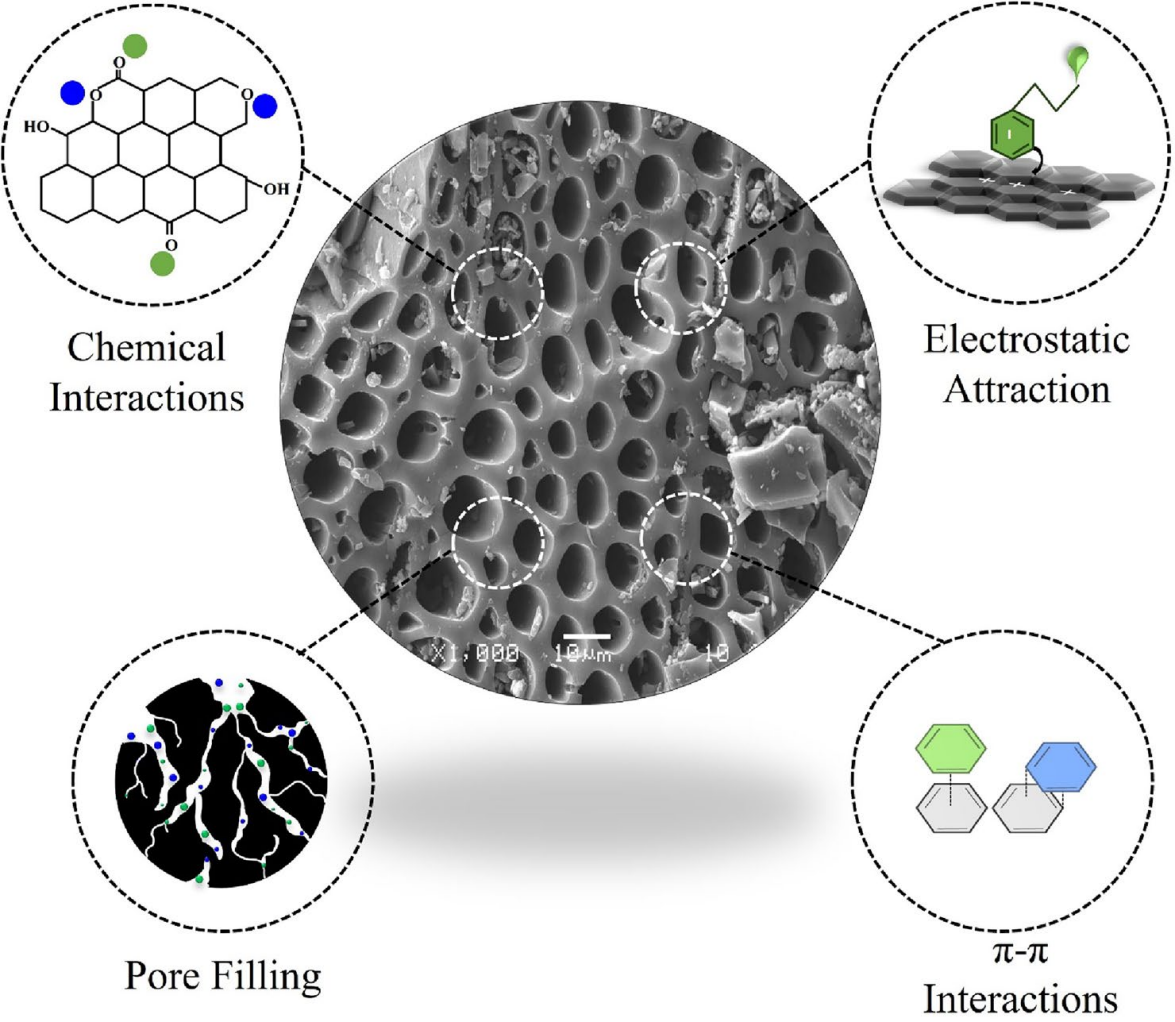
Possible adsorption mechanisms for Microcystin-LR (MCLR) and Saxitoxin (STX) uptake with biochar.

Based on the physicochemical qualities of biochar possess, a number of potential adsorption mechanisms including electrostatic interactions, ion exchange, pore filling, and precipitation, assist to remove organic and inorganic contaminants in aqueous solutions^81^ (Fig. 6). In this study, biochar provided a positively charged porous surface with oxygen containing functionality capable remediating recently high levels of MCLR and STX reported by literature^9^.

Potential pathways for MCLR adsorption with biochar include hydrophobic interactions, hydrogen bonding, electrostatic forces, and π-π interactions. As detailed in the initial pH study, the electrostatic force of attraction is a plausible adsorption mechanism the positively charged biochar interacts with negatively charged MCLR molecules, an attraction is ensued triggering adsorption. As for the hydrogen bonding, the biochar contains substantial amount of acidic oxygen containing function groups of 941.33 ± 0.86 µmol/g (Table S1) which are favorable for chemisorption. These chemical interactions may arise via the polar or polarizable MCLR molecules and the oxygen containing functional groups present on the biochar^69,82^. Hydrophobic interactions play interesting roles for the uptake of MCLR onto biochar. Due to the nature of MCLR in aqueous media, the ADDA group (amino acid groups) responsible for the toxin’s hydrophobicity disallows it from dissolving the in the solution which permits the molecule to adsorb onto the carbon surface of the biochar^69^. As for π–π interactions, biochar presents aromaticity due to low H/C and O/C values (Table S1), and so the delocalized electrons from the biochar form π–π interactions with the delocalized aromatics on the MCLR structure allowing strong attractions. Adsorption mechanisms such as pore filling, have been deemed incapable due to the limitation in pore structure in the biochar as reported by the S_BET_ = 261.06 ± 6.20 m^2^/g, and pore size = 2.49 nm (Table S1). Determined by Zhang et al.^83^, the maximum length and width of the MCLR molecule are 2.94 nm and 2.55 nm respectively, hence the molecule is too bulky to fit in these spaces^71^.

Meanwhile, for STX adsorption with biochar, possible adsorption mechanisms involve both chemical and physical interactions such as dispersive interactions, hydrogen bonding, and pore filling. As discussed, biochar contains favorable acidic oxygen containing groups functional groups which may undergo favorable chemical interactions with the highly polar guanidine groups due to hydrogen bonding^84,85^. Dispersive interactions like Van der Waals interactions are also likely as suggested by literature, due to the instantaneous dipole–dipole interactions between the toxin molecule and the biochar^40^. From the contact time study, earlier reports detailed rapid adsorption occurring between STX and biochar, which connected to a physical pore filling adsorption mechanism. As the biochar contains mesopores, these present accessible spaces for STX uptake as shown in literature^39^. Consequently, from the initial pH study, biochar and STX both shared positive charge which disallowed any electrostatic forces of attraction.

## Conclusions

The biochar provided great adsorption performance for the removal of both MCLR and STX. Observing, key adsorption parameters including dosage amount, contact time, initial concentration and initial pH, high percentage removal were noted under feasible operating conditions. In addition, biochar showed favorable simultaneous adsorption of both toxins in the presence of each other, proving it a useful solution for waters affected by both HAB toxins. The Langmuir–Freundlich model provided a best fit of both toxins, with calculated adsorption capacities of 3507.46 and 622.23 µg/g MCLR and STX respectively.

Analogous to the results, the STX showed best fit behavior for all the investigated kinetic models while MCLR only best fitted model the Elovich model. Thus, providing insight on the occurrence of chemisorption and physisorption adsorption mechanisms between the toxin and the biochar which facilitated high uptake. Thus, these results highlight the exceptional use of biochar for HAB remediation.

For future works, it would be beneficial to investigate the influence of humic acid, coexisting anions, and different waterbodies on the efficiency of adsorption similar to literature^18,19^. Furthermore, the impact of contact time on the adsorption of STX and MCLR onto biochar at different concentrations will be explored. This investigation aims to elucidate how the adsorption rate is affected by varying levels of harmful algal bloom toxins.

### Supplementary Information

Supplementary Information.

## Data Availability

The datasets used and/or analyzed during the current study available from the corresponding author on reasonable request.
