# Supplementary material for: Influence of biochar on the removal of Microcystin-LR and Saxitoxin from aqueous solutions
Source: Sci Rep. 2024 May 14;14:11058. doi: 10.1038/s41598-024-61802-z (PMC11094018; doi:10.1038/s41598-024-61802-z)
Supplement: Supplementary file 1 — Supplementary Information. [file 41598_2024_61802_MOESM1_ESM.docx]

**Supplementary Information**

**Influence of biochar on the removal of Microcystin-LR and Saxitoxin from aqueous solutions**

**Outline**

**Texts**

**Supplementary T1:** Physicochemical and Morphological Characterization of Biochar

**Tables**

Table S1. Ultimate, proximate, and morphological analyses of biochar.

**Figures**

Figure S1. Biochar characterization (a) SEM image X1000, (b) TGA and DTG thermogram and (c) FTIR spectra.

**Supplementary T1**

**2.2 Characterization of Biochar**

Scanning Electron Microscope (SEM) imaging of the biochar was performed using JEOL JSM 6380LV (Tokyo, Japan) under microscope parameters of accelerating voltage of 15 kV, magnification 500x – 2500x and spot size 49. To improve surface imaging, biochar was sputter coated with gold nanoparticles using Denton Vacuum Desk III (Moorestown, MJ). Ultimate analysis of the biochar was performed to obtain the elemental compositions with respect to carbon, hydrogen, nitrogen, and sulfur (wt. %) using a FLASH EA 1112 Series elemental analyzer (Thermo Scientific, Grand Island, NY). Sample preparation and process analysis were strictly adhered to previous studies [1]. Proximate analysis of the biochar was performed via thermogravimetric analysis (TGA) using TGA 4000 (PerkinElmer). Moisture content (MC), and volatile matter (VM) weight percentages were obtained TGA procedure detailed elsewhere [1]. Utilizing the same procedure under combustion conditions (Air flow at 20 mL/min), thermogram (TG) and differential thermogram (DTG) were obtained for the biochar. The ash content analysis was performed using Thermo Scientific Thermolyne Muffle Furnace (Grand Island, NY) under a static air environment set at 575 °C for 5 h. The following equations were used to calculate the moisture, volatile matter, ash, and fixed carbon contents.

Moisture Content = $\frac{\text{M}_{\text{i}}\text{-}\text{M}_{\text{105}}}{\text{M}_{\text{i}}}\text{ ×100\%}$ (1)

Volatile Matter = $\frac{\text{M}_{\text{105}}\text{-}\text{M}_{\text{900}}}{\text{M}_{\text{i}}}\text{ ×100\%}$ (2)

Ash Content = $\frac{\text{M}_{\text{i}}\text{-}\text{M}_{\text{575}}}{\text{M}_{\text{i}}}\text{ ×100\%}$ (3)

Fixed Carbon = 100 – Moisture content – Volatile matter – Ash content (4)

Where M_i_ is the initial mass of the sample, M_105_ is the sample mass at 105 °C, M_900_ is the sample mass at 900 °C, M_575_ is the mass at 575 °C. The fixed carbon (FC) content was calculated by the difference method as shown below subtracting the sum of the moisture content, volatile matter, and ash content.

Brunauer-Emmett-Teller (BET) surface area, total pore volume and micropore volume of the biochar were measured using a High-Pressure Volumetric Analyzer (HPVA II, Norcross, GA) through N_2_ adsorption and desorption experiments. Detailed report of the process is provided by literature [2], which was strictly adhered to for analysis. From the data obtained, Microactive software from Micromeritics Instrument Corporation (Norcross, GA) was used to extrapolate the BET surface area, total pore volume, and micropore volume for the biochar. For the BET surface area, the BET method was applied to the adsorption isotherm with relative pressure (P/P_o_) between 0.05 – 0.35. However, pore volume measurements adsorption data at saturation pressure (P/P_o_) of 0.99 was used to quantify.

For surface functionality analysis, Thermo Scientific Attenuated total reflector FTIR (Model: Nicolet iS5, Madison, WI) was used to identify functional groups present on the biochar. The sample was subject to 64 scans, with 4 cm^-1^ resolution, observed at a wavenumber range of 500 – 4000 cm^-1^. The total acidic oxygen functional group analysis was determined through Boehm Titrations results performed using a Mettler Toledo T50 Titrator (Columbus, OH). The titration process was adopted from literature [3], for the biochar sample. The surface charge of the biochar was determined using surface pH and point of zero charge (pH_PZC_) experiments [4]. The surface pH of biochar was determined by mixing 1 g of the sample with 30 mL of DI water for 24 h. Once complete, the sample was removed using vacuum filtration, and the pH of the filtrate was measured and identified as the surface pH. For the pH_PZC_, in 6 separate flasks 0.25 g of biochar was added, followed by 30 mL of 0.03 M KNO_3_ which were pH adjusted to 2, 4, 6, 8, 10 and 12 using NaOH and HCl, respectively. The mixture was allowed to mix for 24 h at 150 rpm using an automatic benchtop shaker. Once complete, the biochar was removed via vacuum filtration and the pH of the flasks were measured. The pH_PZC_ was extrapolated from a plot with final pH versus initial pH with a tie line, where the intersecting point was the pH_PZC_.

**Results and Discussion**

**Physicochemical and Morphological Characterization of Biochar**

Surface morphology of biochar shows the physical structure etched into the surface due to pyrolysis of the pine biomass (Figure S1a). Figure 2a shows an SEM image of the biochar observed at 1000x magnification. Well defined pore structure of the biochar is shown confirming the high porous nature of biochar. As the biochar is pine derived, a common lignocellulosic biomass [5], tubular pore structure with rich microporosity as shown in Figure S1a. This aligns with findings in literature, such as those reported by Leng et al. in a review article, which suggests that tubular structures are expected in lignocellulosic biomass based biochar leading to a higher microporosity compared to other biomass types [6].

From elemental analysis, biochar reported wt.% of nitrogen, carbon, hydrogen, sulfur, and oxygen 0.88 ± 0.04, 83.61 ± 0.63, 1.69 ± 0.26, BD, and 9.33 ± 0.64, respectively (Table S1). This represents the biochar as a carbon dense material with decreased amount of hydrogen, oxygen, and inorganic elements. The results are consistent with the literature [7-9]. For instance Chen et al. reported bulk elemental content for sawdust-derived biochar that aligns closely with the findings of this study [9]. These results concur a H/C of 0.017 ± 0.004 and an O/C of 0.16 ± 0.05. As an indicator of the biochar carbon structure, the H/C is much less than upper limit 0.7 and the O/C is also less than upper limit 0.4 indicating aromatic ring structures in the biochar [10]. Proximate analysis of the biochar provided in Table S1 complements the elemental analysis as the fixed carbon, volatile matter, moisture content, and ash content wt.% reported at 82.36 ± 0.72, 12.20 ± 0.60, 0.92 ± 0.19, and 4.49 ± 0.07, respectively. Similar, to literature, high fixed carbon with minimal volatile matter, moisture and ash which are the result of the high temperature applied during pyrolysis [11]. Huff et al. also similar values for volatile matter (11.67 wt.%) and fixed carbon (82.11 wt.%) for biochar produced from pinewood at 500 °C [11]. Significant weight loss (%) was observed under combustion temperature conditions (Figure S1b). At temperature of 430 °C, drastic weight loss was displayed similar to literature which accounts to the complex ligneous polymer structure which demands high energy to thermally decompose [12].

From surface porosity experiments of the biochar, BET surface area, total pore volume and micropore volume reported to be 261.06 ± 0.72 m^2^/g, 0.1626 cm^3^/g, and 0.0766 cm^3^/g respectively. These measurements are comparable to literature [13, 14] showing porous nature of pine derived biochar. Igalavithana et al. detailed comparable BET surface area and total pore volume ranging at 53 – 581 m^2^/g and 0.11 – 0.25 cm3/g respectively for pyrolysis performed on pine sawdust at temperatures 300 – 550 °C [13]. While Francisco et al. reported similar results for the micropore volume of commercially obtained pine biochar valued at 0.078 cm^3^/g [14].

Surface functionality experiments showed substantial presence of functional groups present on biochar. From FTIR spectra, minimal peak definition was imaged as shown in Figure S1c. This limitation, is due to the porous nature of the biochar disallowing imaging due to the material absorbing the infrared light [15]. From Boehm titrations results, the acidic oxygen-containing functional groups for the biochar reported as 941.33 ± 0.86 µmol/g comparable to literature as Zhang et al. reported total acidic oxygen containing groups of biochar (barley grass, wheat straw, pine-wood derived), ranging between 910 – 1110 μmol/g [16]. From surface charge experiments, the surface pH and the pH_PZC_ reported at 8.04, and 8.38 respectively, showing slight alkalinity due to the pyrolysis process which discharges alkali salts (from ash content) from the pyrolytic structure [17]. As the pH_PZC_ is greater than the surface pH, the biochar holds a positive surface charge because of the high pyrolytic temperature which reduces the amount of negatively charged functional groups [18].

**References**

[1] C. Chambers, S. Saha, S. Grimes, J. Calhoun, and M. T. Reza, "Physical and morphological alteration of Sargassum-derived ultraporous superactivated hydrochar with remarkable cationic dye adsorption," *Biomass Conv. Bioref.,* 2023, doi: 10.1007/s13399-023-04326-2.

[2] A. I. Sultana, N. Saha, and M. T. Reza, "Upcycling simulated food wastes into superactivated hydrochar for remarkable hydrogen storage," (in en), *Journal of Analytical and Applied Pyrolysis,* vol. 159, p. 105322, 2021/10// 2021, doi: 10.1016/j.jaap.2021.105322.

[3] M. T. Islam, C. Chambers, and M. Toufiq Reza, "Effects of process liquid recirculation on material properties of hydrochar and corresponding adsorption of cationic dye," (in en), *Journal of Analytical and Applied Pyrolysis,* vol. 161, p. 105418, 2022-01 2022, doi: 10.1016/j.jaap.2021.105418.

[4] N. Saha, A. Saba, and M. T. Reza, "Effect of hydrothermal carbonization temperature on pH, dissociation constants, and acidic functional groups on hydrochar from cellulose and wood," (in en), *Journal of Analytical and Applied Pyrolysis,* vol. 137, pp. 138-145, 2019/01// 2019, doi: 10.1016/j.jaap.2018.11.018.

[5] T.-P. Wang *et al.*, "Structures and pyrolytic characteristics of organosolv lignins from typical softwood, hardwood and herbaceous biomass," *Industrial Crops and Products,* vol. 171, p. 113912, 2021/11/01/ 2021, doi: <https://doi.org/10.1016/j.indcrop.2021.113912>.

[6] L. Leng *et al.*, "An overview on engineering the surface area and porosity of biochar," *Science of The Total Environment,* vol. 763, p. 144204, 2021/04/01/ 2021, doi: <https://doi.org/10.1016/j.scitotenv.2020.144204>.

[7] S. Yu, L. Wu, J. Ni, H. Zhang, R. Wei, and W. Chen, "The chemical compositions and carbon structures of pine sawdust- and wheat straw-derived biochars produced in air-limitation, carbon dioxide, and nitrogen atmospheres, and their variation with charring temperature," *Fuel,* vol. 315, p. 122852, 2022/05/01/ 2022, doi: <https://doi.org/10.1016/j.fuel.2021.122852>.

[8] K. Weber and P. Quicker, "Properties of biochar," *Fuel,* vol. 217, pp. 240-261, 2018/04/01/ 2018, doi: <https://doi.org/10.1016/j.fuel.2017.12.054>.

[9] W. Chen, R. Wei, L. Yang, Y. Yang, G. Li, and J. Ni, "Characteristics of wood-derived biochars produced at different temperatures before and after deashing: Their different potential advantages in environmental applications," *Science of The Total Environment,* vol. 651, pp. 2762-2771, 2019/02/15/ 2019, doi: <https://doi.org/10.1016/j.scitotenv.2018.10.141>.

[10] L. Leng, H. Huang, H. Li, J. Li, and W. Zhou, "Biochar stability assessment methods: A review," *Science of The Total Environment,* vol. 647, pp. 210-222, 2019/01/10/ 2019, doi: <https://doi.org/10.1016/j.scitotenv.2018.07.402>.

[11] M. D. Huff, S. Kumar, and J. W. Lee, "Comparative analysis of pinewood, peanut shell, and bamboo biomass derived biochars produced via hydrothermal conversion and pyrolysis," *Journal of Environmental Management,* vol. 146, pp. 303-308, 2014/12/15/ 2014, doi: <https://doi.org/10.1016/j.jenvman.2014.07.016>.

[12] P. Kim *et al.*, "Surface Functionality and Carbon Structures in Lignocellulosic-Derived Biochars Produced by Fast Pyrolysis," *Energy & Fuels,* vol. 25, no. 10, pp. 4693-4703, 2011/10/20 2011, doi: 10.1021/ef200915s.

[13] A. D. Igalavithana *et al.*, "Carbon dioxide capture in biochar produced from pine sawdust and paper mill sludge: Effect of porous structure and surface chemistry," *Science of The Total Environment,* vol. 739, p. 139845, 2020/10/15/ 2020, doi: <https://doi.org/10.1016/j.scitotenv.2020.139845>.

[14] F. J. Lopez-Tenllado, I. L. Motta, and J. M. Hill, "Modification of biochar with high-energy ball milling: Development of porosity and surface acid functional groups," *Bioresource Technology Reports,* vol. 15, p. 100704, 2021/09/01/ 2021, doi: <https://doi.org/10.1016/j.biteb.2021.100704>.

[15] A. I. Sultana, C. Chambers, M. M. N. Ahmed, P. Pathirathna, and T. Reza, "Multifunctional Loblolly Pine-Derived Superactivated Hydrochar: Effect of Hydrothermal Carbonization on Hydrogen and Electron Storage with Carbon Dioxide and Dye Removal," *Nanomaterials (Basel),* vol. 12, no. 20, Oct 12 2022, doi: 10.3390/nano12203575.

[16] Y. Zhang, X. Xu, L. Cao, Y. S. Ok, and X. Cao, "Characterization and quantification of electron donating capacity and its structure dependence in biochar derived from three waste biomasses," *Chemosphere,* vol. 211, pp. 1073-1081, 2018/11/01/ 2018, doi: <https://doi.org/10.1016/j.chemosphere.2018.08.033>.

[17] T. Chen *et al.*, "Influence of pyrolysis temperature on characteristics and heavy metal adsorptive performance of biochar derived from municipal sewage sludge," *Bioresource Technology,* vol. 164, pp. 47-54, 2014/07/01/ 2014, doi: <https://doi.org/10.1016/j.biortech.2014.04.048>.

[18] H. Li, X. Dong, E. B. da Silva, L. M. de Oliveira, Y. Chen, and L. Q. Ma, "Mechanisms of metal sorption by biochars: Biochar characteristics and modifications," *Chemosphere,* vol. 178, pp. 466-478, Jul 2017, doi: 10.1016/j.chemosphere.2017.03.072.

Table S1. Ultimate, proximate, and morphological analyses of biochar.

| **Material Characterization** | **Values** |
| --- | --- |
| **Ultimate Analysis**  Nitrogen  Carbon  Hydrogen  Sulfur  Oxygen  Ash | (wt. %)  0.88 ± 0.04  83.61 ± 0.63  1.69 ± 0.26  BD  9.33 ± 0.64  4.49 ± 0.07 |
| **Proximate Analysis**  Fixed Carbon  Volatile Matter  Moisture Content | (wt. %)  82.36 ± 0.72  12.20 ± 0.60  0.92 ± 0.19 |
| **Porosity**  BET Surface Area (m^2^/g)  Total Pore Volume (cm^3^/g)  Micropore Volume (cm^3^/g)  Pore Size (nm) | 261.06 ± 6.20  0.1626  0.0766  2.49 |
| **Surface Functionality and Charge**  Total Oxygen Functional Groups (Acidic) (µmol/g)  Surface pH  pH_PZC_ | 941.33 ± 0.86  8.04  8.38 |

O: 100 – (N + C + H + S ) BD: Below Detection

H/C = 0.02 O/C = 0.11


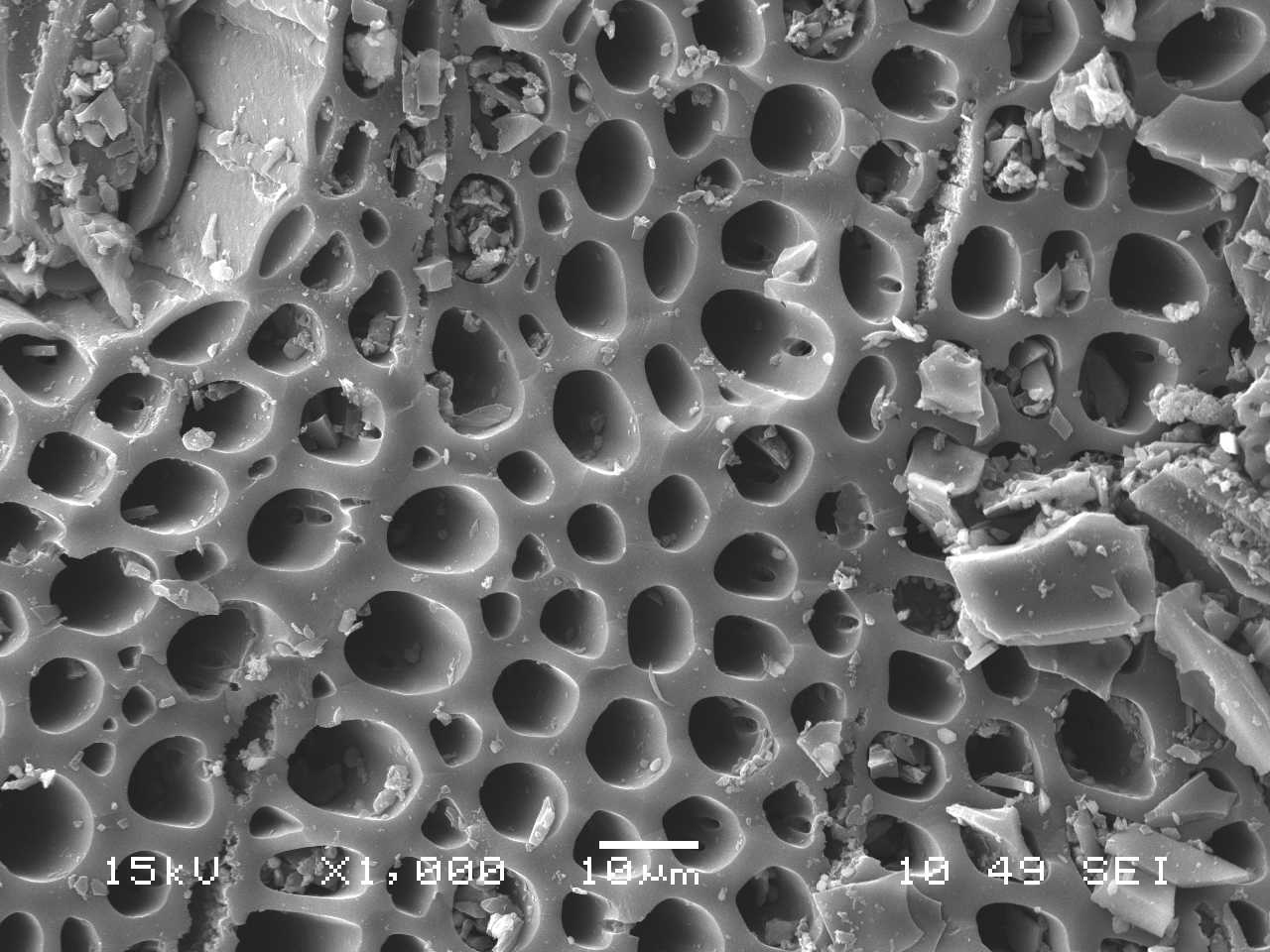


**(a)**

**(b)**

**(c)**

Figure S1. Biochar characterization (a) SEM image X1000, (b) TGA and DTG thermogram and (c) FTIR spectra.
